# Problem-based learning for anesthesia resident operating room crisis management training

**DOI:** 10.1371/journal.pone.0207594

**Published:** 2018-11-19

**Authors:** Nobuyasu Komasawa, Benjamin W. Berg, Toshiaki Minami

**Affiliations:** 1 Department of Anesthesiology, Osaka Medical College, Takatsuki, Osaka, Japan; 2 SimTiki Simulation Center, John A Burns School of Medicine, University of Hawai‘i, Honolulu, Hawaii, United States of America; University of Palermo, ITALY

## Abstract

**Background:**

Senior anesthesia residents must acquire competency in crisis management for operating room (OR) emergencies. We conducted problem based learning (PBL) OR emergency scenarios for anesthesia residents, focused on emergencies in ‘Airway’, ‘Circulation’, ‘Central venous catheter’, and ‘Pain management complications’. Non-technical skills are an integral component of team-based OR emergency management.

**Methods:**

Prior to integrated OR emergency clinical and non-technical skills PBL training, participating 35 anesthesia residents completed two 5-point scale surveys regarding frequency of emergency experiences in the operating room, and self-confidence for anesthesia-related crisis management. Repeat administration of the self-confidence survey was completed immediately following PBL training.

**Results:**

Post-PBL resident clinical management self- confidence improved (P<0.05) in all scenarios on Circulation, Central venous catheter, and Pain treatment related complication topics. Impossible intubation, impossible oxygenation, and awake intubation did not show significant difference following PBL.

**Conclusion:**

Our findings suggest that PBL for OR emergency management can improve resident self- confidence in anesthesia residents.

## Introduction

Advanced life support (ALS) simulation training is a prerequisite for medical staff in critical care specialties [1]. American Heart Association simulation-based ALS courses teach fundamental resuscitation methods for cardiac arrest with a variety of clinical features and dysrhythmias [2]. Compared to ALS content and scenarios perioperative crisis comprises a broader range of life threatening conditions related to factors including airway, circulatory collapse, central venous catheter complications, and pain-treatment related complications, amongst others[3][4][5].

Intraoperative resuscitation requires engagement of a multi-professional pre-formed perioperative team in which an anesthesiologist typically assumes the leadership role. [6]. Typical manikin-based ALS simulation training focuses on individual participant resuscitation skills with limited attention to the non-technical teamwork skills critical for effective intraoperative crisis management [7][8]. Operating room (OR) simulations with adequate fidelity for experiential learning are logistically difficult and resource intensive, and can be delivered to a limited number of participants with each iteration [9],

Problem-based learning (PBL) is an interactive teaching method utilizing a written case stem as learner stimulus for the acquisition and application of cognitive and non-technical skills in emergency clinical settings [10][11]. PBL is one of several established inductive instructional methods, including case based learning, project based learning, and inquiry-based learning [12]. In PBL designed scenarios groups of learners assess and manage a patient described in a scripted scenario based on verbal- and stimulus-triggered feedback received from a facilitator. Well designed PBL cases can simulate OR emergencies and complications related to topics including airway, circulation, central venous catheter, and pain treatment. Manikin based simulation has been used extensively to conduct effective training of skills, knowledge, and teamwork principles related to these clinical topics and others [13]. PBL requires less time and resources than manikin-based in-situ OR emergency scenario training, especially for senior anesthesia residents with relevant clinical experience [14]. A simulation-based healthcare education research agenda published by the Society of Simulation in Healthcare prioritizes Instructional Design including learner characteristics, Outcomes measurement, and Translational research including organizational/systems elements aligned with these research priorities we sought to explore an educational outcome of a PBL simulation based educational method, for a specified learner level, which may require less organizational resources compared to alternative simulation-based strategies [15].

To provide anesthesia resident training in clinical problem solving and non-technical skills for management of life threatening OR critical events, we developed a PBL-based advanced life support in the OR (ALS-Op) curriculum [16]. The curriculum incorporates both knowledge based cognitive medical management, and non-technical skills elements. The primary learning outcome measured was self-confidence. Self-confidence, also called self-efficacy is a critical training outcome, which is necessary, but not sufficient to assure competent application of learned skills and knowledge. Self-confidence is a Kirkpatrick level 2 “learning” outcome. Self-confidence concepts in the modern educational era have been described and extensively studied by Bandura [17][18]. Self-confidence is regularly reported to improve following simulation based training in healthcare [19].

We are unaware of any reports of anesthesia resident self- confidence outcomes following PBL training similar to the ALS-Op curriculum. We sought to evaluate the effect of PBL ALS-Op training on subjective self-confidence of anesthesia residents for management of OR life threatening emergencies.

## Material and methods

Ethical approval was deemed unnecessary by the institutional review board of Osaka Medical College, and participant completion of surveys was considered as consent to the study. We validated this survey by administering an identical PBL and questionnaire to the 5 senior anesthesiologist’s in our department, who provided feedback and supported the content and face validity of the final educational materials.

In Japan, knowledge acquisition through self-directed textbook reading and mentored clinical experiences are the principle methods by which anesthesia residents acquire anesthesia-related emergency management competency.

**Fig 1:**
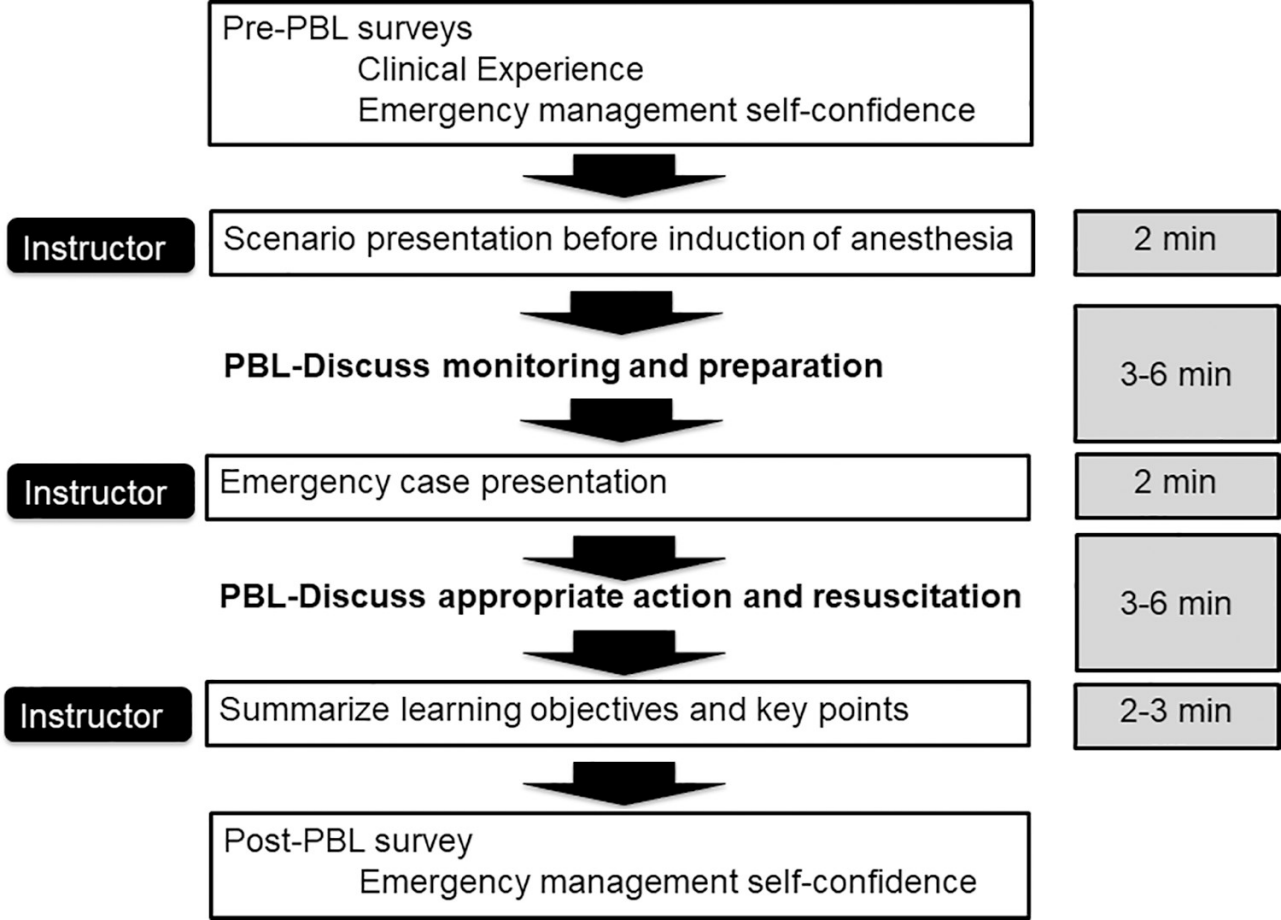
Educational flow diagram and timeline of problem-based learning and discussion.

We developed 23 ALS-Op PBL-based scenarios based on anesthesia-related complication reports [20–25]. Scenario content and the educational flow diagram timeline are shown in Table 1 and Fig 1. Each scenario was presented as a clinical vignette describing the patient, vital signs, and symptoms. Principle scenario content focus areas included problems related to ‘Airway’, ‘Circulation’, ‘Central venous catheter’, and ‘Pain treatment related complications’. Following presentation of each vignette a facilitated interactive session of standardized probing questions and discussion was conducted with the goal of assessing and enhancing resident understanding of the key management principles for each case, including non-technical skills. Multiple standardized ALS-Op PBL cases were sequentially facilitated by the same Japan Society of Anesthesia certified physician anesthesiologist (NK), with certifications by the, American Heart Association Advanced Cardiac Life Support ACLS), and Society of Simulation in Healthcare Certified Healthcare Simulation Educator (CHSE). The content of the scenario was constructed according to the educational guideline of Japanese Society of Anesthesiologists. Individual PBL scenarios were completed in about minutes. A convenience sample of 6–8 residents participated in each ALS-Op PBL session.

**Table 1:** Content of the problem-based learning on advanced life support in the operation room (ALS-OP).

Classification	Theme of the PBL scenarios
Airway 7 scenarios	Impossible oxygenation upon induction of anesthesia
	Impossible intubation upon induction of anesthesia
	Awake intubation for anticipated difficult airway
	Difficult airway management due to anaphylactic shock
	Asthma attack during general anesthesia
	Airway fire during general anesthesia
	Laryngospasm after tracheal extubation
Circulation 6 scenarios	Acute myocardial infarction in the right coronary
	Acute myocardial infarction in the left coronary
	Pulmonary embolism due to deep vein thrombus
	Cardiac arrest due to massive bleeding
	Cardiac arrest due to obstetrics massive bleeding
	Cardiac arrest due to hyperkalemia due to malignant hyperthermia
Central venous catheter (CVC) 5 scenarios	Hypovolemia shock due to subclavian artery damage after CVC
	Obstructive shock due to tension pneumothorax after CVC from internal jugular vein
	Ventricular fibrillation due to guidewire stimulation
	Cardiac tamponade due to guidewire
	Septic shock due to infection by CVC placement
Pain treatment related complications 5 scenarios	Various complications (pneumothorax, late-onset hematoma) after stellate ganglion block
	Accidental intrathecal administration of local anesthetics
	Cardiac arrest due to local anesthetics intoxication
	Serotonin syndrome
	Opioid withdrawal syndrome

Two questionnaires were completed by participants. Before training, a survey was completed regarding participant operating room experience with emergencies which comprised the focus of the ALS-Op PBL curriculum. Frequency of experience was reported on a 5-point scale; `Very Often`, ‘Often’, ‘Occasionally’, ‘Rarely’, and ‘Never’. The 23 PBL case scenarios incorporated content related to each of the clinical experiences included in the experience survey. The second questionnaire was a self-confidence questionnaire completed by participants twice, immediately before, and immediately following ALS-Op PBL training. The self-confidence questionnaire queried the primary the learning objectives for each of the PBL cases. Anesthesia residents reported their self-confidence managing anesthesia-related crises which were the focus of the ALS-Op PBL course on a 5-point scale;

‘Strongly agree’, ‘Agree’, ‘Somewhat agree’, ‘Disagree’, and ‘Strongly disagree’ to the question ‘I feel competent to manage this clinical problem’ [26].

Statistical analysis used chi-square test for comparison of pre- and post-ALS-Op PBL self-confidence for management of operating room anesthesia related crises. P<0.05 was considered statistically significant.

## Results

Between 2014 and 2016, 35 anesthesia residents (24 male and 11 female) with mean clinical experience of 7.2±3.5 years from 6 collaborating teaching hospitals participated in three ALS-Op courses. Each course was facilitated on a single day by a single unique faculty member (NK), and contained identical content with course duration of 6–8 hours, depending on the flow and scripting of interactive dialogue.

**Fig 2:**
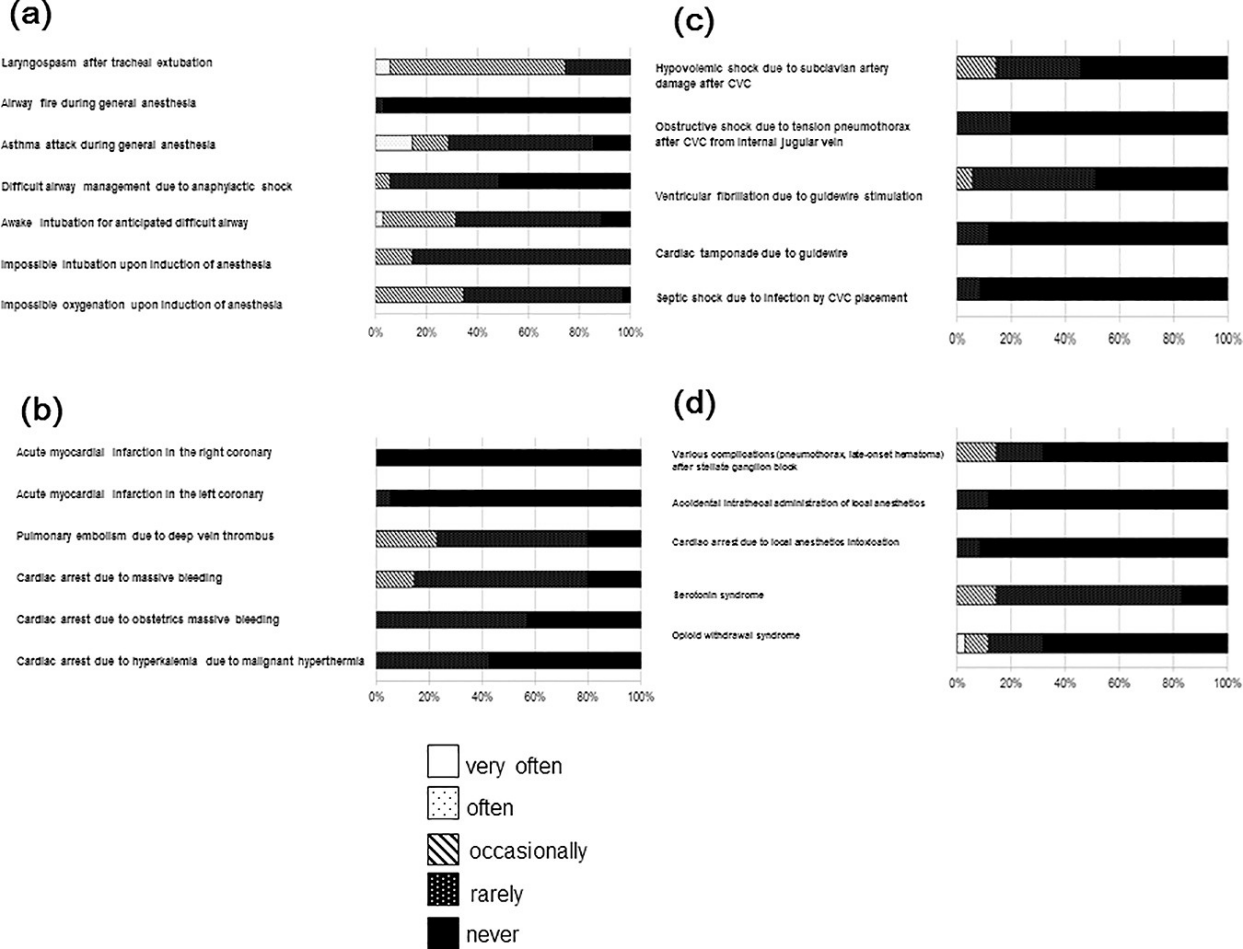
Participants’ answers about several complications they have encountered in the operation room. (a) airway, (b) circulation, (c) central venous catheter, and (d) pain treatment complications.

The primary educational outcome measure for this study was improvement in resident self -confidence following ALS-Op PBL training. Fig 2 shows anesthesia senior resident self-reported historical frequency of clinical experience for each emergency situation. For airway management crisis, all residents had experience managing laryngospasm after tracheal extubation and impossible intubation. 97% of residents had clinical experience managing impossible oxygenation. For circulatory crisis, 80% of residents had managed cardiac arrest due to massive bleeding and pulmonary embolism, while no one has experienced cardiac arrest associated with malignant hyperthermia. For central venous catheter related emergency, 46% of the residents had experienced septic shock, 52% ventricular fibrillation associated with guidewire. 83% of residents had experienced accidental intrathecal administration of local anesthetics as a pain management related complication (S1 Table).

**Fig 3:**
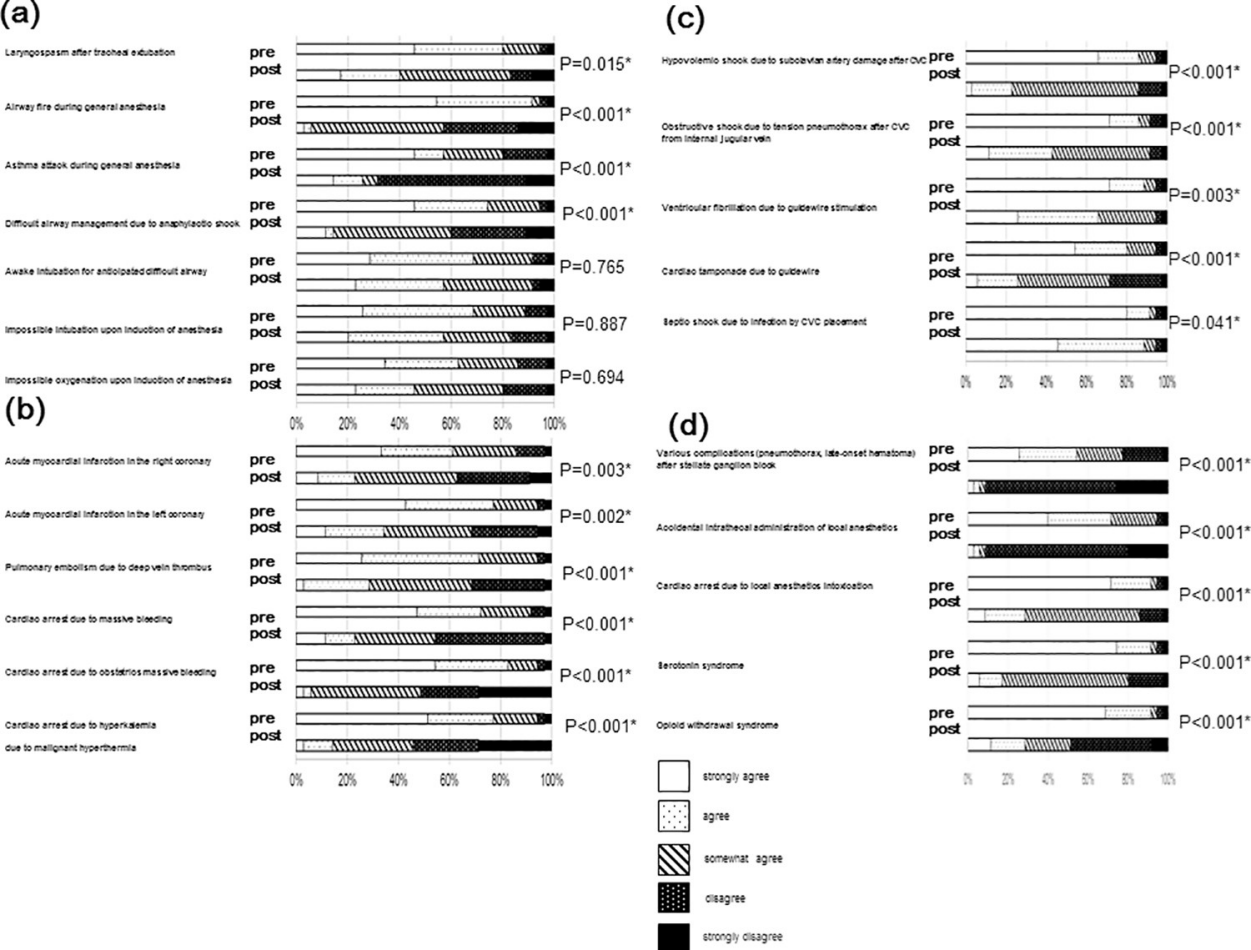
Effect of problem-based learning and discussion in the operation room (OR) toward attitudes to anesthesia senior residents. Comparison of attitudes toward various points of guideline for OR crisis between pre and post course participation. (a) airway, (b) circulation, (c) central venous catheter, and (d) pain treatment complications. Statistical analysis was performed before and after the course. *P<0.05.

Fig 3 shows resident self-confidence regarding management of each clinical emergency. Overall PBL post-course self-confidence combined strongly agree, and agree ratings increased from pre-course ratings in all categories; 35% pre- to 72% post- (P<0.001) in airway, 21% pre- to 80% post- (P<0.001) in circulation, 49% pre- to 86% post- (P<0.001) in CVC, and 17% pre- to 80% post- (P<0.001) in pain management related complications. Impossible intubation, impossible oxygenation, and awake intubation did not show significant differences between pre- and post–PBL (P = 0.694, P = 0.887, and P = 0.765). All other emergency management topics showed significant improvement on the post course self-confidence questionnaire.

## Discussion

Anesthesia resident operating room crisis management and resuscitation skill training is essential for development of core competencies. Intraoperative massive hemorrhage and hypoxia during anesthesia induction are two examples of intraoperative clinical crises which illuminate the imperative for anesthesiologist crisis management and clinical skill competencies [27]. Optimal operating room crisis patient survival rates and prognosis requires that anesthesiologists acquire advanced non-technical skills competency for crisis management including leadership functions [28][29]. We report self-confidence outcomes of an inductive learning strategy. Self-confidence, a necessary but not sufficient component of competency was chosen as an outcome measure in this pilot study to validate the need for further investigation of the PBL approach for training in management of anesthesia emergencies. Self-confidence, (aka self-efficacy) is a foundational theory for simulation based education research [30].

We developed a novel structured PBL approach for perioperative crisis management and advanced life support skills training, incorporating a teamwork component with ALS training. The principle clinical content for ALS-OP included circulation management, airway management, central venous catheters, and pain management-related complications.

ALS-OP instructional design incorporated PBL for two reasons. First is the flexibility of PBL to match learner needs. This approach permits on-the-fly facilitation and scenario modifications to flexibly match the professional needs and level of individual participants [31]. Second, PBL enables simultaneous participation of a larger number of learners, compared to traditional case management and debriefing of manikin-based scenario training [32][33].

Our results demonstrate that resident reported self-confidence for management of impossible intubation, impossible oxygenation, and awake intubation did not show a significant post-training change. We hypothesize that the lack of change in management confidence for these airway specific clinical management challenges is due to the relative greater clinical experience reported for these areas compared to others, with almost 100% of participants reporting some clinical experience in with these emergencies. Emphasis on these specific airway management competencies in previously experienced resident curricular activities, including manikin-based scenario exercises may also account for this finding. In contrast to the lack of improved confidence these three airway competencies, residents reported significant improvement in confidence managing airway problems such as airway fire or laryngospasm after extubation. These contrasting findings suggest that the differential effects of PBL training on self-confidence was primarily related to curricular content elsewhere in residency, and not primarily to clinical experience. Duplicative curricular content may represent a factor to consider when choosing to use PBL; avoidance of duplicative content may allow focusing of PBL on areas in which enhanced self-confidence is a curricular objective. Residents showed significant improvement in self-confidence regarding management of all other focus areas; ‘central venous catheter’, ‘circulation’, and ‘pain related complications’.

We did not specifically measure non-technical skill competencies, but reflect that cultivation of crisis management non-technical skills is challenging and resource intensive using manikin scenario based simulation techniques. In this course we observed active learner engagement in PBL content related to non-technical skill competencies, during a curriculum that integrated clinical management skills.

Simulation in health care is defined in recent literature as “any technology or process that recreates a contextual background and allows a learner to experience success, commit mistakes, receive feedback, and gain confidence in a safe environment” [34][35]. Among the many unique advantages of simulation is the capability to provide experiential exposure to rare operating room clinical crisis events for which clinical management competency is required. PBL in addition to other simulation-based training techniques such as manikin scenarios, can provide anesthesia residents a learner-focused and non-threatening educational environment for learning crisis management, and interactive training for rare life-threatening conditions in the critical care setting such as operating room [36][37].

One limitation of our study is that we did not perform objective evaluation of knowledge or skills regarding anesthesia-related emergency complications [38]. However improvement in the self-confidence outcomes demonstrated in this study suggests that PBL can enhance self-efficacy, an important element of competency development. Self-efficacy is related to competency, as shown in multiple studies [39][40]; “to believe is to achieve”. Objective evaluation of competency in management of anesthesia-related complications is warranted as a next step in evaluating PBL as an instructional technique for anesthesia resident training.

Another limitation is that this study enrolled a relatively small number of participants in a single residency program. Evaluation of ALS-Op training in multiple residency programs with a larger cohort and different curricular experiences will inform the generalizability, define topic areas, and learner levels for optimal PBL implementation strategies for anesthesia resident training.

Finally our study did not directly compare study outcomes for PBL versus alternative educational strategies such as manikin-based scenario or simple lecture. Future simulation-based education research initiatives identified by the Society for Simulation in Healthcare include a focus on System requirements/Challenges including consideration of optimal resource utilization. This study suggests that comparisons of PBL and other simulation methods warrant further investigation regarding both educational outcomes and optimization of organizational resources [15].

This pilot study suggests rigorous assessment of PBL for interprofessional training, a variety of learner levels, defined educational outcomes, and competency in a variety of anesthesia related training domains is warranted.

In conclusion our findings suggest that ALS-OP for PBL can be an effective simulated scenario training methodology. PBL can enhance learning by incorporating a large group problem based learning discussion exercise focused on operating room emergencies with improvement in subjective learner confidence.

## Supporting information

S1 Table Details on participants’ answers about several complications they have encountered in the operation room and effect of problem-based learning and discussion in the OR toward attitudes to anesthesia senior residents. (XLSX)
